# Synthesis and Characterization of Thermoresponsive Chitosan-*graft*-poly(*N*-isopropylacrylamide) Copolymers

**DOI:** 10.3390/polym15153154

**Published:** 2023-07-25

**Authors:** Migle Babelyte, Laura Peciulyte, Vesta Navikaite-Snipaitiene, Joana Bendoraitiene, Volodymyr Samaryk, Ramune Rutkaite

**Affiliations:** 1 Department of Polymer Chemistry and Technology, Kaunas University of Technology, Radvilenu Rd. 19, 50254 Kaunas, Lithuania; migle.babelyte@ktu.edu (M.B.); laura.peciulyte@ktu.edu (L.P.); vesta.navikaite@ktu.lt (V.N.-S.); joana.bendoraitiene@ktu.lt (J.B.); 2 Department of Organic Chemistry, Lviv Polytechnic National University, Stepana Bandery St. 14, 79000 Lviv, Ukraine; volodymyr.y.samaryk@lpnu.ua

**Keywords:** chitosan, *N*-isopropylacrylamide, thermoresponsive copolymers

## Abstract

Thermoresponsive chitosan-*graft*-poly(*N*-isopropylacrylamide) (CS-*g*-PNIPAAm) copolymers of different composition were synthesized by free-radical polymerization of chitosan (CS) and *N*-isopropylacrylamide (NIPAAm) in aqueous solution using potassium persulfate (PPS) as an initiator. By changing the molar ratio of CS:NIPAAm from 1:0.25 to 1:10 graft copolymers with a CS backbone and different amounts of PNIPAM side chains were prepared. The chemical structure of the obtained CS-*g*-PNIPAAm copolymers was confirmed by FTIR and ^1^H NMR spectroscopy. ^1^H NMR spectra were also used to calculate the content of attached PNIPAAm side chains. Moreover, the lower critical solution temperature (LCST) behavior of synthesized copolymers was assessed by cloud point, differential scanning calorimetry and particle size measurements. The aqueous solutions of copolymers containing ≥12 molar percent of PNIPAAm side chains demonstrated LCST behavior with the phase separation at around 29.0–32.7 °C. The intensity of thermoresponsiveness depended on the composition of copolymers and increased with increasing content of poly(*N*-isopropylacrylamide) moieties. The synthesized thermoresponsive chitosan-*graft*-poly(*N*-isopropylacrylamide) copolymers could be potentially applied in drug delivery systems or tissue engineering.

## 1. Introduction

Stimuli-responsive polymers are materials that react strongly to small changes in physical, chemical, or biochemical conditions. These polymers can respond to environmental changes such as temperature, pH, specific ions, light, CO_2_, reducing/oxidizing agents, salts etc. [1,2,3]. The polymers with these properties are also known as “environmentally sensitive”, “intelligent” or “smart” polymers and could be used in nanotechnology, biomedicine, biochemistry and other fields [4,5,6].

One of the most attractive thermoresponsive materials is poly(*N*-isopropylacrylamide) (PNIPAAm) which is highly thermosensitive and shows sharp solubility changes in water around a specific temperature, a temperature that is well known as the lower critical solution temperature (LCST). Its molecules undergo a sharp transition from coil to globule in water around 32 °C, changing from a hydrophilic state below this temperature to a hydrophobic state above it [7]. Above 32 °C, this polymer becomes a precipitate and can be dissolved again by lowering the solution temperature [8]. Due to this phenomenon, PNIPAAm is a promising thermosensitive material as its transition temperature can be adjusted [9]. In order to be used in biological applications, such as drug delivery, a thermoresponsive polymer must possess LCST at a temperature close to the normal human physiological temperature, which is usually around 37 °C. Therefore, suitable synthesis methods should be selected to ensure the control of structural parameters that can influence the phase transition temperature, such as the chemical structure, molecular weight and polymer morphology, e.g., linear, branched, star-shaped, comb, brush, network, or dendrimer [10].

The LCST of PNIPAAm can be changed during the copolymerization with hydrophilic or hydrophobic polymers. Usually, the copolymerization reaction with a hydrophobic polymer can help to decrease the LCST, meanwhile the copolymerization reaction with a hydrophilic polymer can help to increase this temperature [11,12]. Due to its thermoresponsive properties, PNIPAAm can be suitable for biomedical applications such as controlled wound dressings, tissue engineering, and drug delivery systems [13]. However, this polymer has some disadvantages, including limited biodegradability [14,15], low mechanical strength [16], limited drug loading capacity [15], rapid release of active compounds [17], etc. Regarding the low biodegradability of PNIPAAm, its use in clinical practice is limited. A variety of agents and biodegradable polymers, including poly(amino acids), polysaccharides, proteins and synthetic polymers such as poly(esters), poly(caprolactone), and poly(ethylene glycol) could be prospective materials for improving the properties of PNIPAAm. The attached biopolymer or synthetic biodegradable polymer chains could modify the PNIPAAm to effectively increase its biodegradability and biocompatibility [18]. Beyond the latter advantages, the combination with some synthetic or natural biopolymers may provide additional responsivity properties, e.g., pH, magnetic, concentration, etc., and allow for the preparation of dual- and multi-responsive smart delivery materials.

The backbone of the thermoresponsive graft copolymer should be composed of relatively high molecular weight polymer [19]. Therefore, water soluble polysaccharides with biodegradability features are attractive materials for the synthesis of such thermoresponsive graft copolymers. They are attractive natural alternatives to synthetic polymers due to nontoxicity, biocompatibility, etc. In the scientific reports, polysaccharides, such as cellulose [20], carboxymethylcellulose [21], chitosan [10,22,23,24,25,26,27,28], carboxymethyl chitosan [29], dextran [30], and sodium alginate [11,31,32] have been successfully used to obtain graft copolymers with thermoresponsive properties. Though each of these graft copolymers have different chemical compositions, their thermoresponsive behavior is similar, however, variations in LCST can be expected. Such graft copolymer systems may upgrade technological solutions when specific solubility and rheological properties above a given temperature are required in a wide range of biomedical applications, such as tissue engineering [33], drug delivery systems [29] or protein refolding [31].

One of the most promising natural polymers for potential use as a backbone of the thermoresponsive graft copolymer is chitosan, because it is biodegradable, biocompatible, nontoxic and also antimicrobial [34,35,36]. Furthermore, this biopolymer is also pH-sensitive due to the protonation and deprotonation of its terminal amino group [37] and is highly regarded for its use in the fabrication of multi-responsive polymers. Furthermore, chitosan can be easily modified via grafting reactions. Graft copolymerization is a promising technique for the modification of the chemical and physical properties of chitosan and for the broadening of its practical use. A typical chitosan graft copolymer consists of the chitosan backbone and the side chains made of the polymer possessing different properties [19]. Poly(*N*-isopropylacrylamide) is one of the most promising grafted acrylic polymers for a chitosan backbone. Poly(*N*-isopropylacrylamide and CS graft copolymers have been described as potential pH and thermoresponsive materials for drug delivery [10,29] and tissue engineering [33]. Moreover, their capability to increase the oral administration of drugs with low solubility, such as caffeine, naproxen, and paclitaxel, and to promote mucosal administration of hydrophobic drugs have been demonstrated [10]. Furthermore, nano- and microgels of such graft copolymers have been suggested for oncological application in the delivery of bioactive compounds and antibacterial applications; meanwhile, cryogel scaffolds of PNIPAAm and CS have been proposed for bioartificial liver devices and for the purification of plasma [10]. For such applications graft copolymers of chitosan and PNIPAAm containing a large amount of *N*-isopropylacrylamide units in the compositions are usually synthesized [22,23,24,25,26,27,28]. However, there are no data on synthesis and there has been no thermoresponsive behavior investigation of the copolymers covering a broad range of compositions, including those containing low and high contents of PNIPAAm moieties, to clearly estimate the parameters controlling the sharpness and the intensity of the transition, which are crucially important for such applications.

In the present study, seven water soluble, chitosan-*graft*-poly(*N*-isopropylacrylamide) copolymers of different compositions were synthesized by free-radical polymerization of chitosan and *N*-isopropylacrylamide in aqueous solution, and were characterized. The thermoresponsive properties of biobased graft copolymers were evaluated by the means of turbidimetry, differential scanning calorimetry and particle analysis.

## 2. Materials and Methods

### 2.1. Materials

The chitosan (CS) (low molecular weight, M_w_ = 50,000–190,000 Da; the degree of deacetylation 76%) and *N*-isopropylacrylamide (NIPAAm, 97%) were purchased from Sigma-Aldrich (Sigma Chemical Co., St. Louis, MO, USA). Potassium persulfate (PPS, 99%) was purchased from Penta chemicals (Ing. Petr Švec—PENTA, Praha, Czech Republic). All other reagents were of analytical grade and were used as received without further purification.

### 2.2. Synthesis of Chitosan-graft-poly(N-isopropylacrylamide) Copolymers

The chitosan-*graft*-poly(*N*-isopropylacrylamide) copolymers (CS-*g*-PNIPAAm) were synthesized as follows. CS (1% *w*/*v* in the reaction mixture) was dissolved in 1% acetic acid solution, NIPAAm (0.175–7% *w*/*v* in the reaction mixture) was dissolved in water and the solutions were mixed together. The mixture was stirred in a three neck round bottom flask at room temperature for 30 min under a nitrogen atmosphere, then aqueous PPS solution (0.54% *w*/*v* in the reaction mixture) was added, and the temperature was elevated to 60 °C. The polymerization was allowed to proceed for 6 h. After that, the reaction mixture was precipitated into acetone and filtrated to separate the polymeric products. The obtained copolymers were purified by 6 cycles of methanol washing and centrifugation in order to remove PNIPAAm.

### 2.3. Nuclear Magnetic Resonance (NMR) Spectroscopy

^1^H NMR spectra of the samples were recorded with a 400 MHz Bruker^®^Avance III High Resolution spectrometer (Bruker BioSpin GmbH, Rheinstetten, Germany) using D_2_O with acetic acid-d_4_ (Sigma-Aldrich) as a solvent. The solutions (1% *w*/*v*) of CS, PNIPAAm and synthesized copolymer were prepared. Chemical shifts were reported in ppm using the signal of D_2_O (4.79 ppm) as internal standard. To estimate the molar percentages of CS and NIPAAm in the copolymers, the ratio of the integration of the signal at 1.96 ppm corresponding to CS to that of the signal at 1.01 ppm corresponding to PNIPAAm side chains was used. The molar percentages of NIPAAm and CS were calculated as follows:

(1) $$\mathrm{NIPAAm}\%=\frac{\left(\;\frac{H}{n}\cdot 100\right)}{\;\left(\;\frac{H}{n}\right)+\left(\;\frac{A}{3}:0.24\right)}$$

(2) CS%=100%−NIPAAm%

where H is the peak area of the isopropyl group of NIPAAm in the grafted chain, n is the number of isopropyl group protons in NIPAAm, A is the peak area of the acetyl group signal, 3 is the number of acetyl group protons, and 0.24 is the degree of acetylation of CS.

### 2.4. FT-IR Analysis

FT-IR spectra of CS, PNIPAAm and synthesized copolymers were recorded using Frontier spectrophotometer (Perkin Elmer, Inc., Waltham, MA, USA) with a single reflectance horizontal attenuated total reflectance (ATR) cell equipped with a diamond crystal. The data were recorded in the spectral range from 700 to 1550 cm^−1^ by accumulating 5 scans with a resolution of 4 cm^−1^.

### 2.5. X-ray Diffraction Analysis

The X-ray diffraction analysis of CS, PNIPAAm and synthesized copolymers were performed on the D8 Advance diffractometer (Bruker AXS, Karlsruhe, Germany) operating at the tube voltage of 40 kV and tube current of 40 mA. The X-ray beam was filtered with a Ni 0.02 mm filter to select the CuKα wavelength. Diffraction patterns were recorded in a Bragg–Brentano geometry using a fast-counting detector, a Bruker LynxEye, based on a silicon strip technology. The samples were scanned over the range 2θ = 3–70° at a scanning speed of 6° min^−1^ using a coupled two theta/theta scan type.

### 2.6. TG Analysis

TG analysis of CS, PNIPAAm and synthesized copolymers was performed by using TGA 4000 instrument (Perkin Elmer, Inc., Waltham, MA, USA). About 10 mg of dried sample was loaded into the ceramic pan. The measurements were carried out at a heating rate of 10 °C/min under a nitrogen atmosphere. The relevant parameters of thermal decomposition stages were determined using Pyris Data Analysis software (v.10.1.0.0411).

### 2.7. LCST Determination of the Synthesized Copolymer Solutions

The LCST of the PNIPAAm and CS-*g*-PNIPAAm copolymers was determined by cloud point and differential scanning calorimetry (DSC) measurements.

The LCSTs of aqueous and aqueous 1% acetic acid copolymer solutions (0.01 or 0.05% *w*/*v*) were determined from cloud-point measurements: the LCST was estimated as the onset temperature of the increase in optical density (OD) which accompanies phase separation of the polymer. OD measurements were performed using a Cary 60 UV–Vis spectrophotometer. The change in OD was monitored as a function of temperature from 20 to 60 °C, at an interval of 1 °C and at a fixed wavelength of 450 nm. The temperature of the cell holder was controlled with a Varian PCB 1500 temperature controller to an accuracy of ±0.1 °C.

The LCST of the PNIPAAm and CS-*g*-PNIPAAm copolymers was also determined from DSC analysis. A sample of 0.2 mg of PNIPAAm or CS-*g*-PNIPAAm copolymer was weighted and 3.9 mg of distilled water or 1% acetic acid solution was added to an aluminum pan. Differential scanning calorimeter DSC8500 (Perkin Elmer, Inc., Waltham, MA, USA) was used for the experiment in the temperature range from 2 to 45 °C with a heating rate of 10 °C/min under N_2_ gas flow at 20 mL/min. The experiments were repeated twice. The results from the second heating cycle were considered and the temperature of the onset of endothermal peak was used to establish the LCST. Moreover, the enthalpy and peak area were calculated.

### 2.8. Particle Size Analysis

Particle size measurements of the copolymer samples were performed with a Delsa^TM^ Nano C particle size analyzer (Beckman Coulter, Inc., Brea, CA, USA). The Delsa^TM^ Nano C uses photon correlation spectroscopy, which determines particle size by measuring the rate of fluctuations of the laser light intensity scattered by particles. The non-negative least-squares (NNLS) algorithm was used to analyze dynamic light scattering data for the particle size distribution. All measurements of scattered light were made at an angle of 165°. The aqueous and 1% acetic acid aqueous PNIPAAm and CS-g-PNIPAAm solutions (0.01 or 0.05% *w*/*v*) were prepared and tested as a function of temperature from 20 to 50 °C, at an interval of 3 °C. The experiments were carried out in triplicate.

## 3. Results and Discussion

### 3.1. Preparation of Chitosan-graft-poly(N-isopropylacrylamide) Copolymers

**Figure 1:**
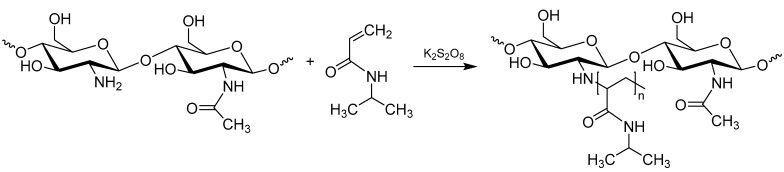
Synthesis scheme of chitosan-*graft*-poly(*N*-isopropylacrylamide).

Chitosan-*graft*-poly(*N*-isopropylacrylamide) (CS-*g*-PNIPAAm) copolymers were synthesized by carrying out free-radical copolymerization of chitosan and *N*-isopropylacrylamide in aqueous solution using potassium persulfate as an initiator as shown in Figure 1.

During this polymerization reaction, the graft copolymers containing chitosan backbone and PNIPAAm side chains were formed. The homopolymer, i.e., PNIPAAm which was possibly formed during the polymerization reaction was removed by the methanol extraction before further analysis. By changing the molar ratio of CS:NIPAAm from 1:0.25 to 1:10 the copolymers of different compositions were prepared. The reaction conditions, feed molar ratio and copolymer compositions are given in Table 1.

**Figure 2:**
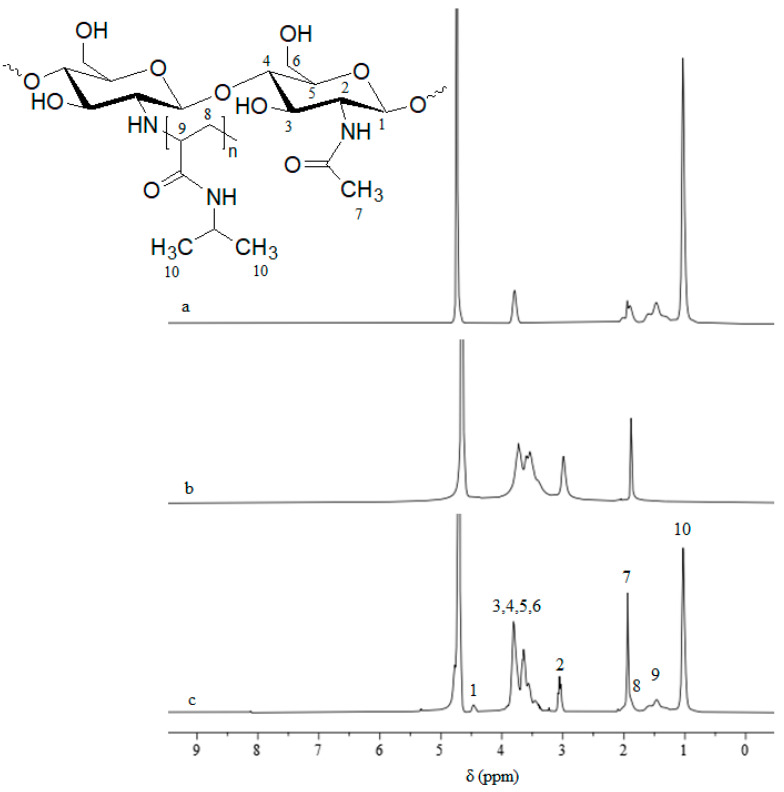
^1^H-NMR spectra of PNIPAAm (**a**), CS (**b**) and CS-*g*-PNIPAAm-4 (**c**).

The synthesized copolymers were characterized by ^1^H-NMR spectroscopy to confirm the structure of synthesized copolymers. The ^1^H-NMR spectra of CS, PNIPAAm and CS-*g*-PNIPAAm-4 are presented in Figure 2. It can be seen that the signals at 1.01 ppm (signal 10), 1.59 ppm (signal 8) and 1.46 ppm (signal 9) corresponding to the respective CH_3_, CH_2_, CH protons in the PNIPAAm moiety [25] are present in the copolymer spectrum. Meanwhile, the signal centered at 1.96 ppm (signal 7) corresponds to CH_3_ protons in acetyl group and the peak at 4.79 ppm originates from the D_2_O solvent [32]. Additionally, the peak 4.49 ppm (signal 1) corresponds to H_1_ and the peak 3.03 ppm (signal 2) corresponds to H_2_ of the pyranose repeating unit of CS_._ Moreover, the peaks from 3.23 to 3.81 ppm (signals 3, 4, 5, 6) are assigned to H_3–6_ of the pyranose repeating unit in CS [26]. These results clearly indicate the introduction of PNIPAAm side chains onto the chitosan backbone.

^1^H-NMR spectroscopy was also used to determine the content of NIPAAm and CS in the synthesized copolymers, except the CS-g-PNIPAAm-7, the solubility of which was not sufficient to run the NMR. The ratio of the integrations of the signal at 1.96 ppm corresponding to acetyl group of CS to those of the signal at 1.01 ppm corresponding to the isopropyl group of the NIPAAm was considered and the weight percentages of NIPAAm and CS in the copolymers were calculated (see Table 1). It can be noted that the amount of NIPAAm units introduced into the graft copolymer was 2–3 times lower compared with that in the reaction feed and ranged from 7.3 to at least 37.3 mol%. Nevertheless, the graft copolymers containing substantial content of PNIPAAm chains have been synthesized.

### 3.2. Characterization of Graft Copolymers

#### 3.2.1. FT-IR Analysis

**Figure 3:**
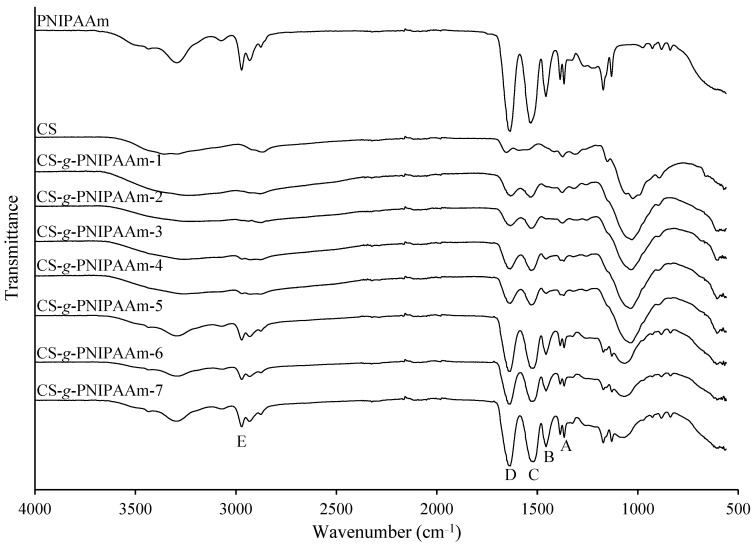
FT-IR spectra of PNIPAAm, CS and CS-*g*-PNIPAAm copolymers.

FT-IR spectra were recorded to confirm the structure of synthesized copolymers. The FT-IR spectra of PNIPAAm, CS and synthesized copolymers (CS-*g*-PNIPAAm) with feed molar ratio of CS:NIPAAm ranging from 1:0.25 to 1:10 are shown in Figure 3. The characteristic bands at 2970 cm^−1^ (peak E) and 1456 cm^−1^ (peak B) corresponding to the C–H stretching and bending vibrations, respectively, of the –CH group were observed in the spectrum of PNIPAAm and CS-*g*-PNIPAAm copolymers. In addition, in the spectra of PNIPAAm and CS-*g*-PNIPAAm copolymers a doublet (peak A) at 1367 cm^−1^ and 1386 cm^−1^ can be assigned to the C–H bending vibrations of –CH(CH_3_)_2_ group. In the spectra of CS-*g*-PNIPAAm the intensity of amide I and amide II bands at 1638 cm^−1^ (peak D) and 1516 cm^−1^ (peak C) [22], respectively, were increased compared with the absorbance peaks in the CS spectrum. Moreover, by the increasing content of NIPAAm units in the copolymers, the intensity of characteristic peaks at 1638 cm^−1^ (peak D) and 1516 cm^−1^ (peak C) corresponding to C=O and N–H vibrations, respectively, were also increasing. The observed spectral changes undoubtedly confirm the formation of the graft copolymer with different content PNIPAAm chains.

#### 3.2.2. X-ray Diffraction Analysis

**Figure 4:**
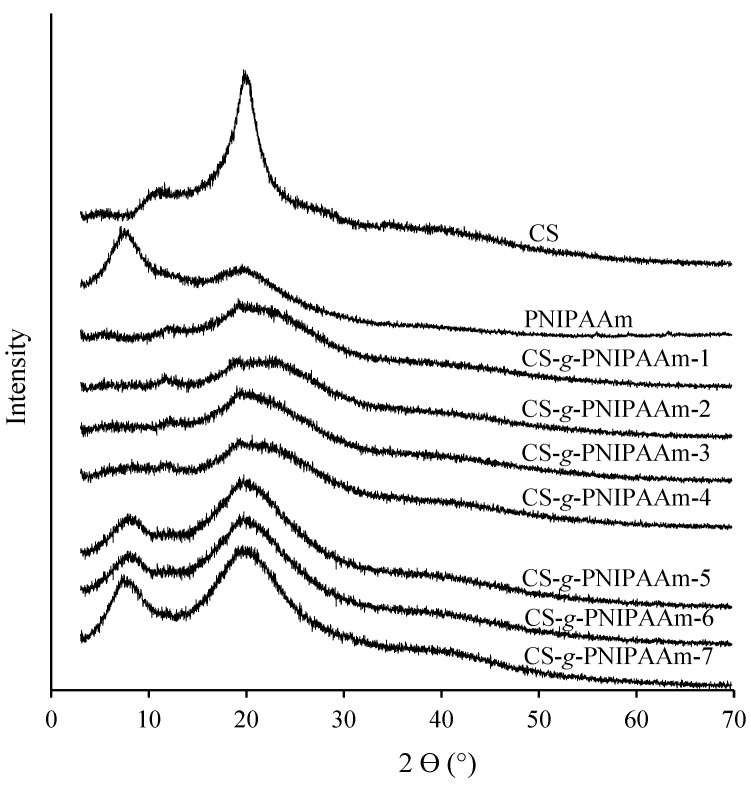
Diffractograms of CS, PNIPAAm and CS-*g*-PNIPAAm copolymers.

The structure of prepared CS-*g*-PNIPAAm copolymer samples was also studied by XRD analysis. The X-ray diffraction spectra of CS, PNIPAAm and synthesized copolymers are shown in Figure 4. The diffractogram of chitosan exhibited two characteristic peaks at 11.3 and 20.1° which confirmed the semi-crystalline state of chitosan [38]. The peaks around 11.3° and 20.1° are associated with the crystal-I and crystal-II structures of chitosan, respectively, and both of these peaks confirm a quite high degree of crystallinity [39]. Kim et al. have showed that the X-ray diffraction pattern of PNIPAAm has two diffraction peaks at 7.7° and 19.8° which are also obvious in the spectrum of PNIPAAm, presented in Figure 4. The broad diffraction peak of PNIPAAm at 19.8° is the result of intermolecular interactions, while the peak at 7.7° is the consequence of the interaction of the polymer chain due to the presence of bulky side groups, which clearly confirms the amorphous state [28].

As can be seen in the diffractograms of graft copolymers containing low amounts of PNIPAAm i.e., CS-*g*-PNIPAAm-1, CS-g-PNIPAAm-2, CS-g-PNIPAAm-3 and CS-g-PNIPAAm-4, the intensity of the peaks at 20.1° decreased drastically in comparison to chitosan. According to Kurita et al. [40] this is due to the introduction of the bulky PNIPAAm side chains into the CS backbone and the breaking down of the crystal structure. Meanwhile, peaks at 7.7° and 19.8°, characteristic of PNIPAAm, were present in the diffractograms of graft copolymers with high content of PNIPAAm moieties i.e., CS-g-PNIPAAm-5, CS-g-PNIPAAm-6 and CS-g-PNIPAAm-7. Although the peak at 19.8° is also visible in the spectra of CS-*g*-PNIPAAm-1, 2, 3, 4 copolymers, the intensity of this peak is much lower compared with that in the spectra of the CS-g-PNIPAAm-5, 6, 7 samples. The obtained X-ray diffraction analysis clearly indicates that the crystalline regions of chitosan were decreased by the graft polymerization and attachment of PNIPAAm side chains.

#### 3.2.3. TG Analysis

**Figure 5:**
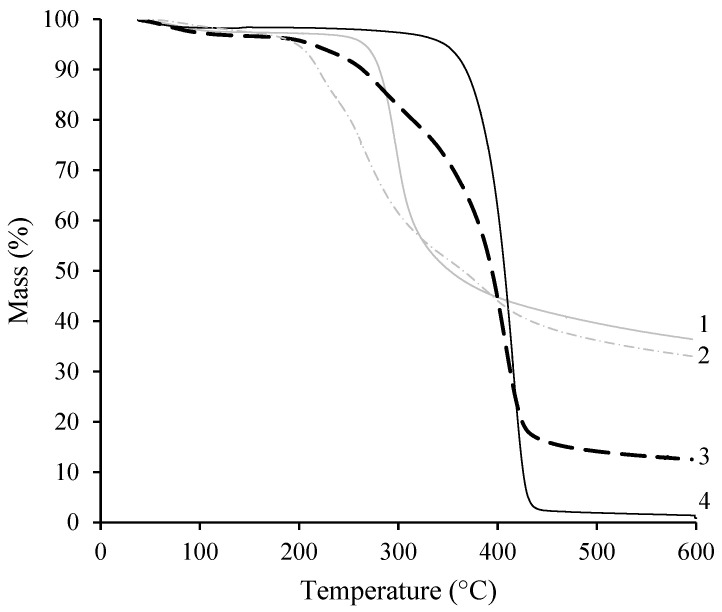
TGA curves of CS (1), CS-*g*-PNIPAAm-4 (2), CS-*g*-PNIPAAm-6 (3) and PNIPAAm (4).

The thermograms of CS, PNIPAAm and CS-*g*-PNIPAAm copolymers were recorded and thermal degradation of CS-*g*-PNIPAAm copolymers was compared with those of the relevant homopolymers (Figure 5, Table 2). One step degradation was characteristic of CS and PNIPAAm—PNIPAAm showing higher thermal stability than CS—until complete degradation at around 438 °C (see Figure 5, curve 4). Degradation of PNIPAAm was confined to a single degradation step starting at 371 °C. The thermogram of CS exhibited degradation starting at 277 °C and was due to the dehydration of the pyranose ring, to depolymerization, and to the decomposition of acetylated and deacetylated units [41]. Meanwhile, the two-step degradation process was characteristic of synthesized copolymers (Table 2). The first step of significant weight loss of the synthesized copolymers at 200–247 °C corresponds to the decomposition of the CS backbone. The second major weight loss step starting at 254–386 °C is related to the decomposition of PNIPAAm grafts. Whereas PNIPAAm showed higher thermal stability than CS, the copolymers with high content of PNIPAAm (CS-g-PNIPAAm-5, 6, 7) exhibited a first degradation step at 236–247 °C and second degradation step at 373–386 °C. Meanwhile, the copolymers (CS-g-PNIPAAm-1, 2, 3, 4) with lower content of NIPAAm exhibited first degradation at 200–203 °C and second degradation at 254–260 °C.

Due to the high amount of carbon atoms in CS and in the copolymers containing high content of CS, and because the measurements were carried under a nitrogen atmosphere, the residual mass in the case of those samples was quite high. However, it was observed that the synthesized copolymers of different composition had a different mass residue, and with the increasing content of PNIPAAm grafts in the copolymer, the residual mass decreased. This confirmed the presence of CS in all of the copolymers, as PNIPAAm was almost completely decomposed at this temperature.

### 3.3. Termoresponsive Behavior of Chitosan-graft-poly(N-isopropylacrylamide) Copolymers

#### 3.3.1. Determination of LCST of the Copolymer Solutions

As described earlier, the LCST is defined as the temperature at which a macromolecule undergoes a coil-to-globule transition [7]. Below the LCST, hydrogen bonding between water molecules and the hydrophilic amide groups of the PNIPAM occurs. This results in the formation of water clusters around the polymer, which induces a flexible open coil conformation. Hence, the polymer is soluble. Above the LCST, hydrogen bonds between the macromolecule and water are weakened. Thus, the water clusters break, promoting closer association of the hydrophobic isopropyl groups. This has the effect of changing the polymer chain conformation from a relaxed coil to a tight globule. The polymer becomes insoluble and, consequently, a phase separation occurs [7,8].

**Figure 6:**
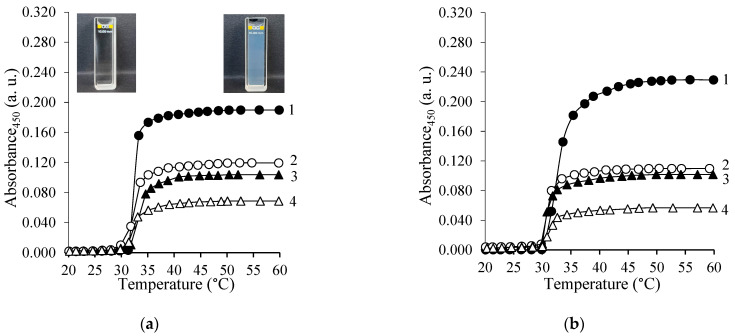
Optical density of aqueous (**a**) and aqueous 1% acetic acid (**b**) solutions of PNIPAAm and CS-*g*-PNIPAAm as a function of temperature: PNIPAAm (1), CS-*g*-PNIPAAm-7 (2), CS-*g*-PNIPAAm-6 (3), CS-*g*-PNIPAAm-5 (4). The polymer concentration 0.01% *w*/*v*.

**Figure 7:**
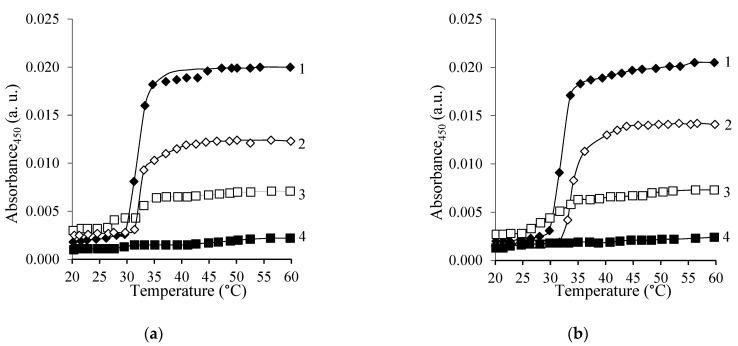
Optical density of aqueous (**a**) and aqueous 1% acetic acid (**b**) solutions of PNIPAAm and CS-*g*-PNIPAAm as a function of temperature: CS-*g*-PNIPAAm-4 (1), CS-*g*-PNIPAAm-3 (2), CS-*g*-PNIPAAm-2 (3), CS-*g*-PNIPAAm-1 (4). The polymer concentration 0.05% *w*/*v*.

Due to the thermoresponsive nature of PNIPAAm it was important to also evaluate the properties of the synthesized copolymers and their behavior in aqueous medium. Therefore, first, the thermoresponsive behavior of copolymers in aqueous solutions was assessed by cloud point measurements. Figure 6a and Figure 7a show the changes in the absorbance of PNIPAAm and CS-*g*-PNIPAAm aqueous solutions at 450 nm by changing the temperature from 20 to 60 °C. The optical density increases with the increase of the temperature, rising sharply in the narrow temperature range of 29.7–31.6 °C, which is defined as the LCST. The curves in Figure 6a and Figure 7a, and the data presented in Table 3, clearly show that the LCST of the samples is very similar, and that LCST behavior is characteristic of graft copolymers containing ≥12 mol% of PNIPAAm side chains. However, by increasing the content of PNIPAAm in the synthesized copolymers the amplitude increases, showing the effect of PNIPAAm content on the intensity of the thermoresponsive coil to globule transition.

Similarly, the thermoresponsive behavior of CS-*g*-PNIPAAm was also assessed in aqueous acetic acid solutions due to the better solubility of the synthesized copolymers. It was revealed that aqueous acetic acid solutions of copolymers containing ≥12 mol% of PNIPAAm grafts exhibit LCST behavior i.e., sharp solubility change in aqueous acetic acid solution at around 29.2–30.6 °C (see Figure 6b and Figure 7b and Table 3). The amplitude of those changes was also dependent on PNIPAAm content and on copolymer concentration. The slightly lower LCST values found for the PNIPAAm and CS-*g*-PNIPAAm copolymers in the aqueous acetic acid solution could be due to reactions to the different media and pHs of the solutions [42].

**Figure 8:**
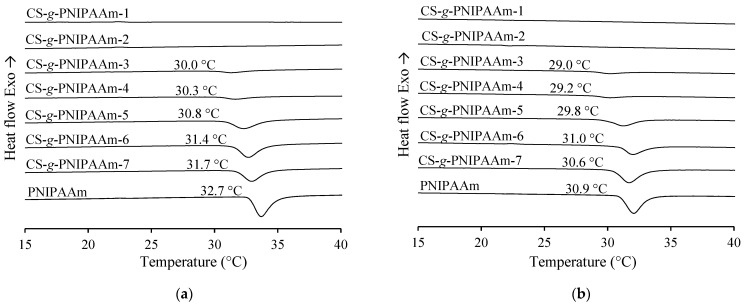
DSC thermograms of PNIPAAm and CS-*g*-PNIPAAm aqueous (**a**) and aqueous 1% acetic acid (**b**) solutions with the indicated LCST values.

The LCST of the PNIPAAm and CS-*g*-PNIPAAm copolymers in aqueous and aqueous acetic acid solutions was also determined from the DSC thermograms. The results from the second heating cycle were reported and the temperature of onset of endothermal peak was defined as the LCST [28]. The data indicate that the LCST behavior was typical of PNIPAAm and copolymers containing ≥ 18.3 mol% of PNIPAAm grafts, namely, CS-*g*-PNIPAAm-3, 4, 5, 6, 7. The LCST of PNIPAAm and CS-*g*-PNIPAAm copolymers was around 30.0–32.7 and 29.0–30.9 °C depending on the solution medium (see Figure 8 and Table 3). It can be observed that, by increasing the content of PNIPAAm in the synthesized copolymers, the endothermal peak area and enthalpy also increased. This is in good agreement with cloud point measurement data and indicates the effect of PNIPAAm content on the intensity of thermoresponsive properties.

To conclude, the LCST determined from DSC and cloud point measurements were very similar. However, the values were not exactly the same because of the differences in methodology and sensitivity among the two different methods.

#### 3.3.2. Particle Size Analysis

**Figure 9:**
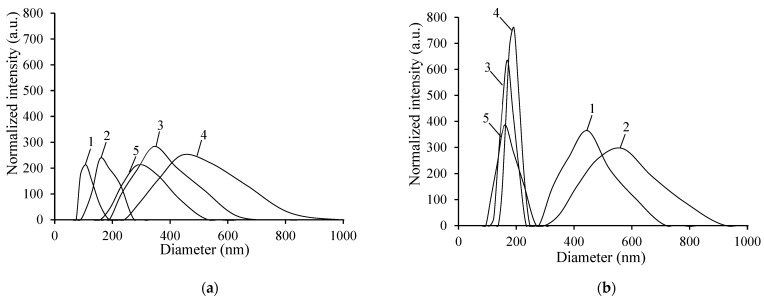
Particle size distribution of copolymers in aqueous acetic acid solutions at the temperatures of 20 (**a**) and 45 °C (**b**): 1—CS-*g*-PNIPAAm-3; 2—CS-*g*-PNIPAAm-4; 3—CS-*g*-PNIPAAm-5; 4—CS-*g*-PNIPAAm-6; 5—CS-*g*-PNIPAAm-7. Particle size measurements of CS-*g*-PNIPAAm-3 and CS-*g*-PNIPAAm-4 were performed at a copolymer solution concentration of 0.05% *w*/*v*. Particle size measurements of CS-*g*-PNIPAAm-5, CS-*g*-PNIPAAm-6 and CS-*g*-PNIPAAm-7 were performed at a copolymer solution concentration of 0.01% *w*/*v*.

In order to understand the conformational transitions of copolymers in aqueous or acetic acid solutions, the particle size of thermoresponsive copolymers was analyzed at temperatures below and above LCST (see Table 4 and Figure 9).

**Figure 10:**
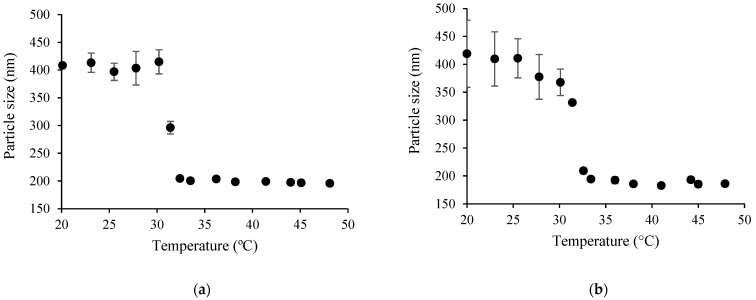
Average particle diameter of copolymers in water as a function of temperature: CS-*g*-PNIPAAm-5 (**a**); CS-*g*-PNIPAAm-6 (**b**).

**Figure 11:**
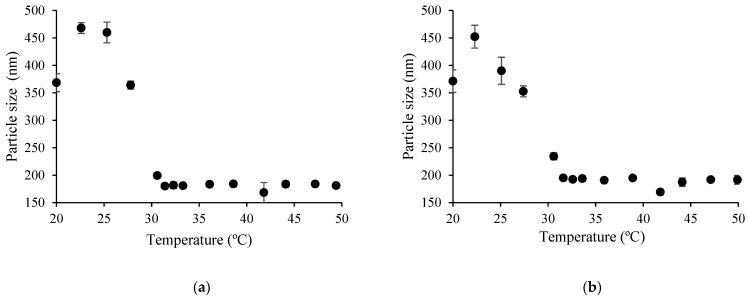
Average particle diameter of copolymers in aqueous 1% acetic acid solution as a function of temperature: CS-*g*-PNIPAAm-5 (**a**); CS-*g*-PNIPAAm-6 (**b**).

As can be seen from the data presented in Table 4 and Figure 9, the diameter of copolymer coils or particles in the tested medium depended strongly on the content of PNIPAAm moieties in the graft copolymers. When the amount of PNIPAAm in the CS-*g*-PNIPAAm-3,4 copolymers was 18.3–20.5 mol%, the size of copolymer particles at 45 °C (above the LCST) was higher than the size of copolymer coils in the solution at 20 °C. However, by having a higher content of PNIPAAm (≥25.5 mol%) in the CS-*g*-PNIPAAm-5, 6, 7 copolymers, an opposite behavior was observed. As can be seen from Table 4 and Figure 9 as well as from Figure 10 and Figure 11, the particle size of copolymers above the LCST was lower than that at 20 °C. This can be attributed to dehydration of PNIPAAm chains, which generates a more compact structure due to a hydrophobic interaction between PNIPAAm hydrophobic groups [43].

A similar dependency on PNIPAAm content particle size vs. temperature behavior has also been observed by other authors. In the paper of Vasile and Nita [31], the alginate-*g*-PNIPAAm copolymers showed a reduction in particle size with increased temperature, while in the case of low PNIPAAm content, a contrary behavior was observed. This phenomenon was also determined by other authors [44] when the aggregation of formed nanoparticles caused by PNIPAAm polymer chain interaction occurred above the LCST, leading to an increase of the measured particle size. In addition, Yang et al. [45] have suggested that the transition of two types can occur when changing the temperature of the system containing poly(*N*-isopropylacrylamide) and its copolymers. When a short-chain copolymer is obtained, the system shows a phase transition from a small particle size (segmented phase) to a large particle size (aggregated phase). On the contrary, when a long-chain copolymer is obtained, the particle size decreases (collapsed state) by increasing the temperature.

In the present work, the particle size of PNIPAAm in the 0.01% *w*/*v* solutions was about 154 ± 29 nm below LCST, meanwhile the particle size was increased up to 470 ± 42 nm above LCST. Therefore, it can be presumed that, in the case of synthesized PNIPAAm and graft copolymers with a low content of PNIPAAm (18.3–20.5 mol%), shorter PNIPAAm side chains were formed. Meanwhile, in the case of PNIPAAm content in the copolymer ≥ 25.5 mol%, longer PNIPAAm side chains were attached to the CS backbone. Meanwhile, the particle size of CS in aqueous acetic acid solution was about 248 ± 34 nm and PDI was 0.46 ± 0.05, which is quite similar to copolymer PDI below LCST. Furthermore, it can be noted that the particle size vs. temperature curves presented in Figure 10 and Figure 11 resemble very well the cloud point measurement curves.

## 4. Conclusions

Graft copolymers of chitosan and *N*-isopropylacrylamide were successfully synthesized via radical polymerization in aqueous medium using potassium persulfate as an initiator. By changing the molar ratio of CS:NIPAAm from 1:0.25 to 1:10 we prepared seven graft copolymers with chitosan backbone and PNIPAAm side chains. The formation of poly(*N*-isopropylacrylamide) grafts was confirmed by X-ray diffraction analysis, ^1^H-NMR and FTIR spectroscopy. Furthermore, ^1^H-NMR spectroscopy was used to determine the content of *N*-isopropylacrylamide and chitosan units in the synthesized copolymers. Moreover, the aqueous solutions of almost all synthesized copolymers demonstrated LCST behavior with phase separation at around 29.0–32.7 °C as determined by calorimetric, turbidimetric and light scattering methods. It was revealed that the amplitude of thermoresponsiveness depended on the composition of copolymers and increased with increasing content of poly(*N*-isopropylacrylamide) moieties. The thermogravimetric analysis demonstrated that, by increasing the content of PNIPAAm in the synthesized copolymers, the thermal stability also increased. The obtained data will serve as basic information for the synthesis of fluorescently labelled PNIPAAm and CS graft copolymers, which will allow further studies of the behavior of those copolymers by applying various steady-state and time-resolved fluorescence spectroscopy methods. Finally, the studies of chitosan-*graft*-poly(*N*-isopropylacrylamide) copolymers are of great importance for the potential applications of such materials in the biomedical field.

## Figures and Tables

**Table 1:** Reaction conditions and composition of synthesized copolymers.

Copolymer Sample	Molar Ratio of Reagents			Comonomer Molar Ratio in Feed, %		Reaction Yield, %	Comonomer Molar Ratio in Copolymer, %	
	CS	NIPAAm	PPS	CS	NIPAAm		CS	NIPAAm
CS-*g*-PNIPAAm-1	1	0.25	0.16	80.0	20.0	55.2	92.6	7.4
CS-*g*-PNIPAAm-2	1	0.5	0.16	66.7	33.3	53.9	88.0	12.0
CS-*g*-PNIPAAm-3	1	0.8	0.16	55.6	44.4	55.1	81.7	18.3
CS-*g*-PNIPAAm-4	1	1	0.16	50.0	50.0	57.2	79.5	20.5
CS-*g*-PNIPAAm-5	1	3	0.16	25.0	75.0	57.8	74.5	25.5
CS-*g*-PNIPAAm-6	1	5	0.16	16.7	83.3	63.6	62.7	37.3
CS-*g*-PNIPAAm-7	1	10	0.16	9.1	90.9	77.3	-	-

**Table 2:** Characteristic parameters of thermogravimetric analysis.

Sample	Decomposition Onset Temperature, °C		Residual Mass, %
	First Step	Second Step	
CS	277	-	36.4 ± 0.2
PNIPAAm	371	-	0.8 ± 0.3
CS-*g*-PNIPAAm-1	201	254	36.2 ± 0.2
CS-*g*-PNIPAAm-2	203	258	35.8 ± 0.3
CS-*g*-PNIPAAm-3	200	258	35.4 ± 0.2
CS-*g*-PNIPAAm-4	203	260	33.0 ± 0.4
CS-*g*-PNIPAAm-5	238	373	12.9 ± 0.3
CS-*g*-PNIPAAm-6	236	379	12.5 ± 0.2
CS-*g*-PNIPAAm-7	247	386	7.0 ± 0.4

**Table 3:** Cloud point and DSC measurement results.

	In Aqueous Solution			In Aqueous 1% Acetic Acid Solution		
Sample	LCST, °C (Cloud Point)	LCST, °C (DSC)	Enthalpy, J/g	LCST, °C (cloud point)	LCST, °C (DSC)	Enthalpy, J/g
CS-*g*-PNIPAAm-1	-	-	-	-	-	-
CS-*g*-PNIPAAm-2	29.7 ^a^	-	-	29.2 ^a^	-	-
CS-*g*-PNIPAAm-3	30.9 ^a^	30.0	5.41	30.2 ^a^	29.0	5.12
CS-*g*-PNIPAAm-4	31.0 ^a^	30.3	6.36	29.9 ^a^	29.2	5.32
CS-*g*-PNIPAAm-5	31.0 ^b^	30.8	25.74	30.1 ^b^	29.8	21.10
CS-*g*-PNIPAAm-6	31.6 ^b^	31.4	33.81	30.0 ^b^	31.0	23.86
CS-*g*-PNIPAAm-7	31.0 ^b^	31.7	29.32	30.0 ^b^	30.6	35.83
PNIPAAm	31.4 ^b^	32.7	44.26	30.6 ^b^	30.9	43.65

^a^—cloud point measurements were performed at a copolymer solution concentration of 0.05% *w*/*v*. ^b^—cloud point measurements were performed at a (co)polymer concentration of 0.01% *w*/*v*.

**Table 4:** Particle size and PDI of copolymers in water at the temperatures of 20 and 45 °C.

Copolymer Sample	Particle Diameter, nm	PDI	Particle Diameter, nm	PDI
	20 °C		45 °C	
CS-*g*-PNIPAAm-3 ^a^	164 ± 98	0.34 ± 0.11	634 ± 215	0.27 ± 0.07
CS-*g*-PNIPAAm-4 ^a^	267 ± 46	0.20 ± 0.08	554 ± 114	0.16 ± 0.03
CS-*g*-PNIPAAm-5 ^b^	408 ± 1	0.31 ± 0.04	197 ± 10	0.15 ± 0.04
CS-*g*-PNIPAAm-6 ^b^	419 ± 60	0.31 ± 0.07	185 ± 5	0.07 ± 0.04
CS-*g*-PNIPAAm-7 ^b^	383 ± 43	0.44 ± 0.06	157 ± 15	0.20 ± 0.09

^a^—particle size measurements of the samples were performed at a copolymer solution concentration of 0.05% *w*/*v*. ^b^—particle size measurements of the samples were performed at a copolymer solution concentration of 0.01% *w*/*v*.

## Data Availability

Not applicable.
